# Phylogeny, Diversification, and Biogeography of *Garra* (Cypriniformes: Cyprinidae) Reveals Multiple Cross‐Drainage Dispersals in Southeast Asia

**DOI:** 10.1002/ece3.70448

**Published:** 2024-11-17

**Authors:** Zhi‐Bang Wang, Thaung Naing Oo, Lan‐Ping Zheng, Xiao‐Yong Chen

**Affiliations:** ^1^ Southeast Asia Biodiversity Research Institute, Chinese Academy of Sciences, Yezin Nay Pyi Taw Myanmar; ^2^ State Key Laboratory of Genetic Resources and Evolution and Yunnan Key Laboratory of Biodiversity and Ecological Conservation of Gaoligong Mountain Kunming Institute of Zoology, Chinese Academy of Sciences Kunming China; ^3^ Yunnan International Joint Laboratory of Southeast Asia Biodiversity Conservation Mengla China; ^4^ University of Chinese Academy of Sciences Beijing China; ^5^ Department of Forest Ministry of Natural Resources and Environmental Conservation Nay Pyi Taw Myanmar; ^6^ College of Chinese Materia Medica, Yunnan University of Chinese Medicine Kunming China

**Keywords:** biogeography, *Garra*, phylogeny, species delimitation

## Abstract

*Garra*, a kind of small‐ to medium‐sized fish, is widely distributed from southern Eurasia to central Africa. As one of the most diverse genera of Cyprinidae, investigating the phylogeny and biogeography of *Garra* remains challenging. In this study, we combined sequences of *Garra* samples collected from Myanmar with sequences downloaded from GenBank to investigate the phylogeny, diversification, and biogeography of *Garra* on a global scale, with an emphasis on Southeast Asia. Species delimitation results indicated that there are at least 22 potential species, including eight undescribed species in Myanmar, suggesting that the diversity of *Garra* in this region have been largely underestimated. Diversification analysis suggested a relatively high diversification rate in the early branches of *Garra*. Ancestral distribution reconstruction results revealed that *Garra* originated from the Irrawaddy River basin in the late Eocene, approximately 34 million years ago, with subsequent dispersals across different drainages influenced by the uplift of the Qinghai‐Tibet Plateau. Our study provided a new insight into the evolutionary history of *Garra* and the basis for further research on this genus.

## Graphical abstract

*Garra*, a small‐ to middle‐sized freshwater fish, is one of the most diverse genera within Cyprinidae. Investigating the phylogeny and biogeography of *Garra* under a comprehensive species sampling framework remains challenging, due to the lack of Southeast Asian species, which has hindered our understanding of the evolutionary history of *Garra*. In this study, we conducted analyses by combining sequences of *Garra* samples collected from Myanmar with published sequences to investigate phylogeny, diversification, and biogeography of *Garra* on a global scale, with a specific focus on Southeast Asia. Our results revealed that *Garra* was originated from the Irrawaddy River basin with subsequent dispersals across different drainages influenced by the Qinghai‐Tibet Plateau uplift.

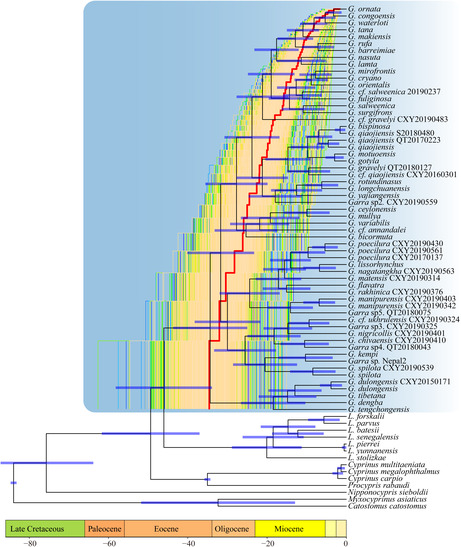

## 1 Introduction

Species of the genus *Garra* are small‐ to middle‐sized freshwater fishes that mainly feed on algae (Zhang, Yue, and Chen 2000). The most outstanding morphological character of *Garra* is the mouth structure that evolved into an adhesive disc to adapt to water flow (Thoni, Gurung, and Mayden 2016). To date, over 180 species have been described in the genus, *Garra* is considered as one of the most diverse genera within Cyprinidae (Tangjitjaroen et al. 2023).

The distribution of *Garra* ranges from southern China, Southeast Asia, South Asia, and Middle East to central Africa (Sun et al. 2018; Zhang and Chen 2002). Over the past decades, numerous research has been devoted to investigating the taxonomy and biogeography of this genus. Presently, originating from Southeast Asia and extending distribution range from Asia to Africa through multiple dispersal events have been widely evidenced (Menon 1964; Tang, Getahun, and Liu 2009; Yang and Mayden 2010; Zheng, Yang, and Chen 2012). However, comprehensive species sampling remains challenging, resulting in only broadly depicted phylogenetic and biogeographic patterns.

The taxonomic history of *Garra* is intricate, marked by unstable taxonomic status within the species. Several reasons result in this complexity. Primarily, early described species were often simplistic (Nebeshwar and Vishwanath 2013, 2017), focusing on general traits and mouth structures. Many *Garra* species exhibit similar feeding habits and living environment, and convergent adaption could lead to morphological similarity. Consequently, researchers might struggle to accurately identify species. Species described in early stage were typically attributed with extensive distribution ranges (Menon 1964; Nebeshwar and Vishwanath 2013). However, species highly adapted to flowing water usually present restricted ranges (Kottelat 2001). Therefore, widely recorded *Garra* species should be reevaluated because they may potentially contain multiple distinct species (Nebeshwar and Vishwanath 2013).

Within this study, we collected *Garra* species from Myanmar to fill the gap in Southeast Asian species data. With more Southeast Asian species included, we aim to comprehensively investigate the phylogeny, evolutionary rates, and biogeography on a global scale, but mainly focus on Southeast Asia, hoping to provide a new insight into the evolutionary history of *Garra* and facilitate further research on this genus.

## 2 Materials and Methods

### 2.1 Taxon Sampling and DNA Sequencing

Specimen were collected from the Irrawaddy River basin, Salween River basin, and Kaladan River basin across Myanmar during 2014 ~ 2019. Detailed information of sampling sites is provided in Figure S1.

Genomic DNA were extracted from right pelvic fin clips preserved in 95% ethanol using DNA Isolation Mini Kit (Vazyme Biotech Co., Ltd). We amplified and sequenced three mitochondrial genes: cytochrome oxidase subunit I (*COI*), cytochrome *b* (*CYTB*), and 16S ribosomal RNA (*16S*), and four nuclear genes: recombination activating gene 1 exon 3 (*RAG1*), rhodopsin (*RH*), interphotoreceptor retinoid binding protein gene (*IRBP*), and early growth response protein 2B gene (*EGR2B*). The primers and experimental protocols followed Yang et al. (2012).

### 2.2 Phylogeny Construction and Species Delimitation

For the initial phylogenetic tree construction, all sequences of sampled species were employed for phylogeny construction by concatenated dataset. PartitionFinder2 (Lanfear et al. 2017) was used to infer the most suitable models for each codon position partition under Akaike information criterion corrected (AICc). Maximum likelihood (ML) tree was constructed in IQ‐TREE v2.2.0 (Minh et al. 2020) with 100,000 ultrafast bootstrapping.

To identify potential species, we firstly checked the morphology of species following Kullander and Fang (2004), Qin et al. (2017), and Nebeshwar and Vishwanath (2017), and then we conducted species delimitation using five methods including Automatic Barcode Gap Discovery (ABGD), Assemble Species by Automatic Partitioning (ASAP), General Mixed Yule Coalescent (GMYC), Bayesian Poisson tree processes (bPTP), and multi‐rate Poisson tree processes (mPTP). ABGD, while originally designed for single‐locus data (Puillandre et al. 2021), proved effective for multi‐locus data as well (Arrigoni et al. 2016). Here, concatenated dataset and ML tree generated from combined dataset were used for species delimitation. Based on delimitation results, each identified potential species were reduced to one concatenated sequence, except *G*. *qiaojiensis* QT20180223 and *G. qiaojiensis*. S20180480 were also included, for following analysis. The default parameters were used in all delimitation methods. The final conclusion of species delimitation was based on molecular and morphologic delimitation results.

We also downloaded sequences from GenBank to include the major distribution areas of *Garra* and meanwhile to balance the sequences quality and proportion of missing sequence. Finally, a total of 48 species (including 14 outgroups) were downloaded for phylogeny construction that covered almost all the major distribution area of *Garra*. Downloaded sequences and species list are provided in Table S1. Combining dataset of identified potential species, sequences of each gene were aligned by MAFFT v7.313 (Katoh and Standley 2013) and checked manually for inconsistencies. Then, the seven gene sequences matrixes were concatenated in PhyloSuite v1.2.2 (Zhang et al. 2020). The most suitable models of each gene partitions were identified using PartitionFinder v2 (Lanfear et al. 2017) under Akaike information criterion corrected (AICc). The suggested most suitable models of each partition are given in Table 1.

**TABLE 1:** Partitions and substitution models identified by PartitionFinder.

	Partition	Length (bp)	Substitution model
Mitochondrial	*COI*_pos1	283	TIM + I + G
	*COI*_pos2	283	HKY + I + G
	*COI*_pos3	283	GTR + I + G
	*CYTB*_pos1	363	SYM + I + G
	*CYTB*_pos2	363	HKY + I + G
	*CYTB*_pos3	363	GTR + I + G
	1*6S*	619	GTR + I + G
Nuclear	*RAG1*_pos1	488	TVM + I + G
	*RAG1*_pos2	488	GTR + I + G
	*RAG1*_pos3	488	SYM + G
	*IRBP*_pos1	271	TIM + I + G
	*IRBP*_pos2	271	GTR + I + G
	*IRBP*_pos3	271	SYM + G
	*RH*_pos1	273	GTR + I + G
	*RH*_pos2	273	GTR + I + G
	*RH*_pos3	273	GTR + G
	*EGR2B*_pos1	271	HKY + I + G
	*EGR2B*_pos2	271	HKY + I + G
	*EGR2B*_pos3	271	TVM + G

Maximum likelihood tree was inferred using IQ‐TREE2 with 100,000 ultrafast bootstrapping. The Maximum clade credibility (MCC) tree was constructed in BEAST v1.10.4 (Suchard et al. 2018) with 160,000,000 chain lengths and 1000 sampling frequency. The effective sample sizes of all parameters were ensured by larger than 200.

### 2.3 Divergent Time Estimation and Diversification Analysis

The divergent time was estimated in BEAST v1.10.4. Two calibration points were set following Gu et al. (2024). The first calibration point is the divergence between Catostomidae and Cyprinidae, of which the median time is 97 MYA. We used the lognormal distribution with parameters setting as follows: mean = 2.1, standard deviation = 0.4, and offset = 81.9. The second calibration point is the separation between *Procypris* and *Cyprinus*, *Carassioides*, and *Carassius* based on the oldest *Procypris*‐like cyprinid fossil from the late Eocene (Chen et al. 2015). We used the lognormal distribution with parameters setting as follows: mean = 1.2, standard deviation = 0.4, and offset = 33.9. Chain length was set to 50,000,000 with a sampling frequency 1000. Three independent runs were performed. To make sure that all the effective sample size larger than 200, an additional run with 10,000,000 chain length was performed. The outputs of all runs were combined and then checked in Tracer v1.7.2 to ensure the effective sample size of each parameter larger than 200. A consensus tree was generated from the output of three runs with the first 10% samples as burn‐in TreeAnnotator.

We performed diversification rate estimation in Bayesian Analysis of Macroevolutionary Mixtures (BAMM) based on time‐calibrated phylogeny (Rabosky 2014). Four independent Markov Chain Monte Carlo (MCMC) chains with chain length as 10,000,000 generations were run with sampling frequency as 1000. Priors was set with the R package BAMMtools (Rabosky et al. 2014) using a conservative prior of one rate shift. The number of described *Garra* is over 180 and still presents a huge increase in potential. Given this extensive diversity, it is impossible to cover all described species. Consequently, we tested three sample fractions, which were 0.328, 0.295, and 0.236, assuming the total species numbers of 180, 200, and 250. This test aimed to assess potential speciation rate change across different proportions of included taxa. The convergence of each MCMC run was confirmed. Furthermore, we performed Lineage throughout time (LTT) plot using APE v5.6‐2 package (Paradis and Schliep 2019) in an R environment to visualize the rate of lineage increase over the evolutionary timescale of *Garra*.

### 2.4 Ancestral Distribution Area Reconstruction

We first categorized the distribution areas into five areas including South China, Southeast Asia, South Asia, Middle East, and Africa. The detailed distribution and code could be found in Table S2. The ancestral distribution area reconstruction was performed in RASP v4.3 (Yu, Blair, and He 2020) with the time‐calibrated phylogenetic tree as input. The best fitting model was chosen based on the result of Model test function. Because of the restrict distribution range of *Garra* species, the maximum link area was set to two. Considering the high proportion of samples with detailed distribution information in Southeast Asia, we further categorized the distribution areas by river drainage for species from this area. Other species were categorized based on regions, such as Southern China, Middle East, South Asia, and Africa. The analysis steps were consistent with before. The jump dispersal model (+J parameter) was under debate as it tends to be overestimated in models with this parameter (Ree and Sanmartín 2018). We do not use the +J parameter when comparing models considering the weak dispersal ability of *Garra* species.

## 3 Results

### 3.1 Data Matrix and Species Delimitation

**FIGURE 1:**
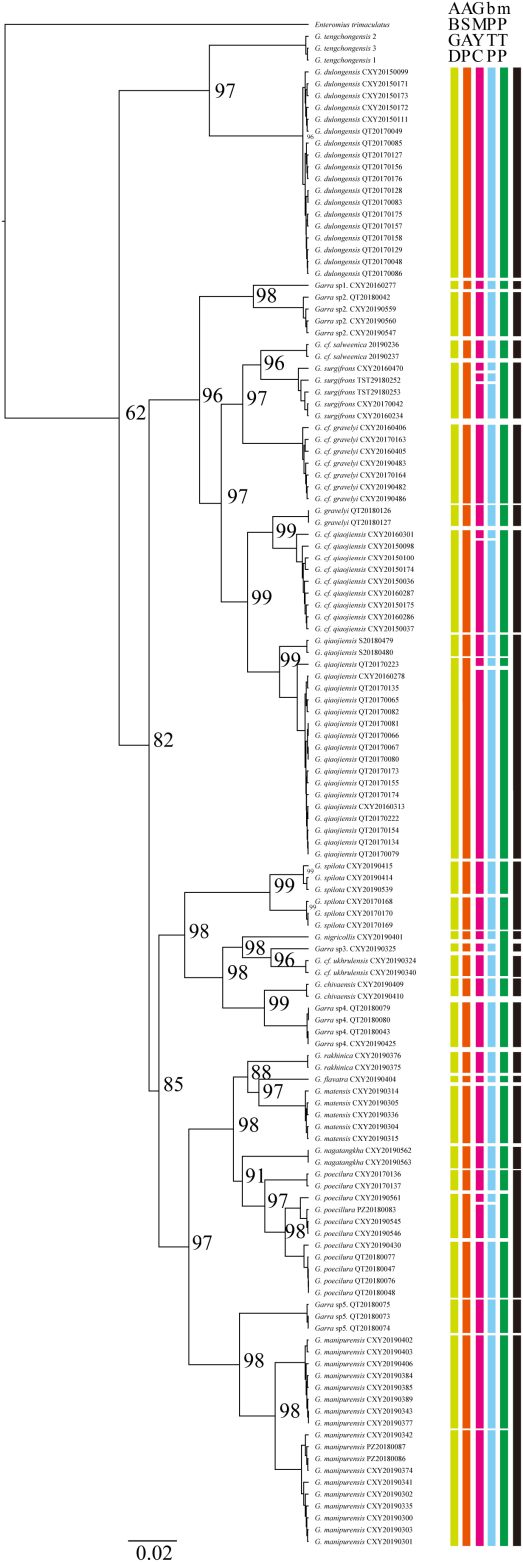
Maximum likelihood phylogenetic tree of Myanmar samples based on concatenated dataset of three mitochondrial genes and four nuclear genes. Colored bars denote the results from different species delimitation methods, yellow: ABGD, orange: ASAP, red: GMYC, blue: BPTP, green: MPTP, and black: Finial species delimitation decision. Values on the branch represent ultrafast bootstrap values for main nodes.

The concatenated dataset contains 6466 bp from 129 individuals (*COI*: 615 bp from 125 individuals; *CYTB*: 984 bp from 129 individuals; *16S*: 544 bp from 129 individuals; *RAG1*: 1450 bp from 121 individuals; *RH*: 804 bp from 128 individuals; *IRBP*: 778 bp from 123 individuals; *EGR2B*: 782 bp from 125 individuals). The detailed information of samples and sequences could be found in Supporting Information S1. The main nodes of ML tree were well supported (UFbootstrap 62 ~ 99) (Figure 1).

Various species delimitation methods yield similar results with minor conflicts in some branch. A total of 26 ~ 32 species were identified based on different molecular delimitation methods (Figure 1). By comprehensively encompassing morphology and distribution information, the finial number of potential species was reserved as 22 including eight species that were identified as undescribed species.

### 3.2 Reduced Phylogeny, Divergent Time Estimation, and Diversification Analysis

**FIGURE 2:**
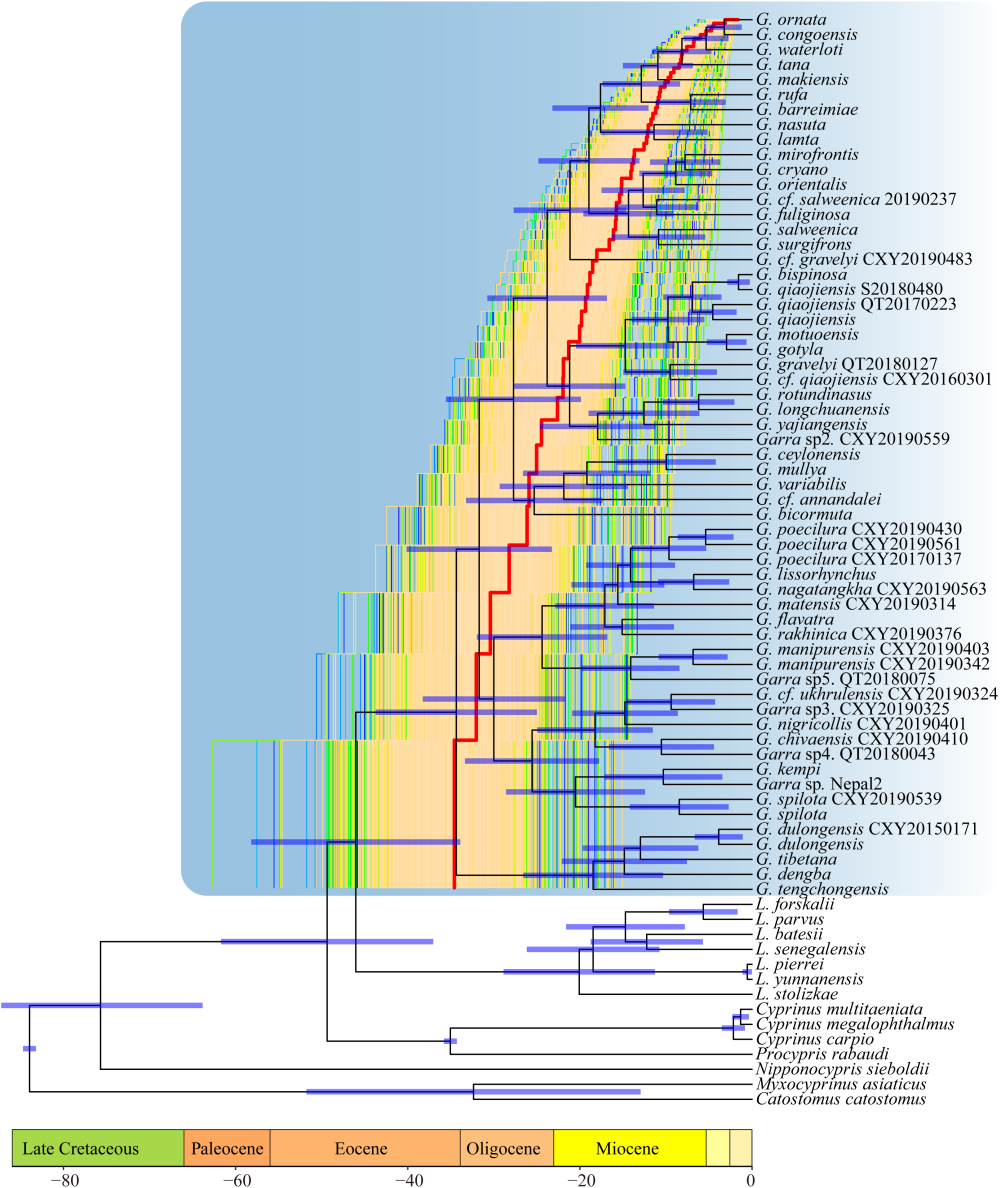
Divergent time estimation and lineage‐throughout‐time (LTT) plot. The blue background indicates the *Garra* lineages. The purple bar on each node denotes the 95% highest posterior density. The LTT plot illustrates the cumulation lineage curve of *Garra* with current species coverage, where the red line denotes the MCC tree, and other lines denote randomly sampled 5000 trees generated in BEAST.

**FIGURE 3:**
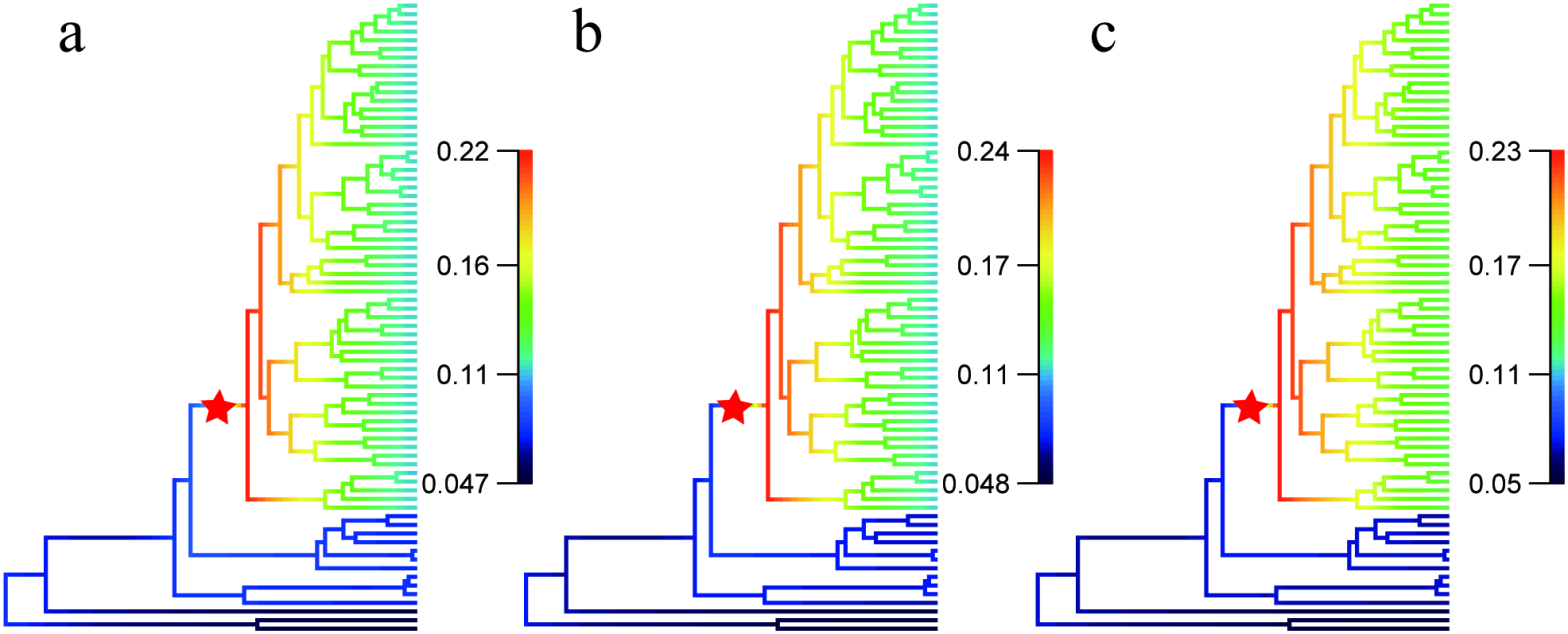
Diversification rate analysis based on time‐calibrated phylogenetic tree. The colors on the branches represent the mean of the posterior density of diversification rate, with red stars indicating the *Garra* branch. Three different conditions were considered, assuming varying total species numbers: (a) 180 species, (b) 200 species, and (c) 250 species.

The two methods yielded similar results. The MCC tree was used for calibration. Divergent time estimation indicated that the most recent common ancestor of *Garra* originated from late Eocene, approximately 34 million years ago (MYA) (Figure 2). The LTT plot revealed that the accumulation of lineages of *Garra* increased by a relatively constant rate after origination, followed by a decrease in the rate of accumulation around 12.6 MYA to the present (Figure 2). All simulations of BAMM analysis showed no significant rate change across the diversification of *Garra*. With the assuming total number of *Garra* increased, there is no marked speciation rate change. The diversification rate initially remained relatively high in early branches, gradually decreasing to the present (Figure 3).

### 3.3 Ancestral Area Reconstruction

**FIGURE 4:**
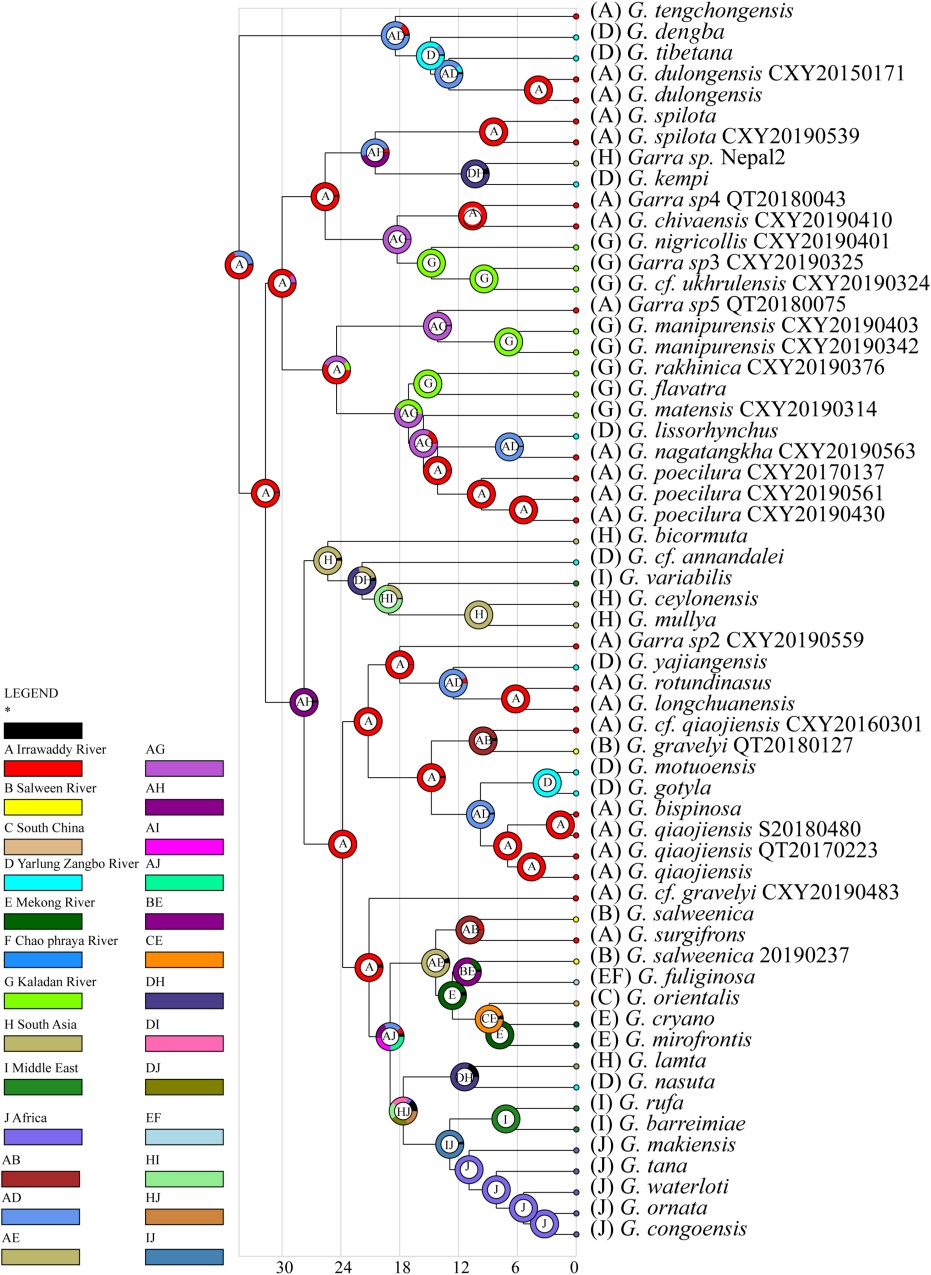
Reconstruction of ancestral distribution of *Garra*. Based on the current *Garra* species distributions, 10 river basins or regions were assigned. A: Irrawaddy River basin, B: Salween River basin, C: Southern China, D: Yarlung Zangbo River basin, E: Mekong River basin, F: Chao Phraya River basin, G: Kaladan River basin, H: Indian Peninsular, I: The Middle East, J: Africa, and *: Unknown. The pie chart on each node represents different possibilities of distributions, with the letter in the middle of the pie chart indicating the distribution with the highest possibility.

According to the result of model selection, DIVALIKE was the best‐fitted model (Table 2). Reconstruction of ancestral distribution indicated that the common ancestor of *Garra* originated in the Southeast Asia (Figure S2). With more detailed categories, the result suggested that the common ancestor of *Garra* originated in the Irrawaddy River basin, with multiple cross‐drainage dispersals observed (Figure 4). These dispersals mainly happened between the Irrawaddy River and neighboring river basins, most frequently with the Yarlung Zangbo River. Additionally, reverse dispersals were identified between the Irrawaddy River and the Yarlung Zangbo River or the Kaladan River basin.

**TABLE 2:** Result of biogeographic model test. The best model is highlighted in bold.

Models	LnL	Numbers of parameters	Parameters		AICc	AICc_wt
			*d*	*e*		
DEC	−74.1	2	0.0030	152.4	152.4	0.0059
**DIVALIKE**	**−68.97**	**2**	**0.0037**	**142.2**	**142.2**	**0.99**
BAYAREALIKE	−87.63	2	0.0025	0.027	179.5	7.8e‐09

## 4 Discussion

*Garra*, being one of the most diverse genera within Cyprinidae, has drawn the attention of numerous ichthyologists who are dedicated to studying its taxonomy and evolutionary history. However, many efforts have concentrated on local taxonomy and discovery of cryptic species, only a limited number of studies discussed the phylogenetic relationships and biogeographic history of *Garra* within the framework of Labeoninae (Yang and Mayden 2010; Yang et al. 2012).

### 4.1 Phylogeny and Diversity of Myanmar *Garra*

Our phylogeny was consistent with previous studies that *Garra tengchongensis* as the oldest lineage of *Garra* (Yang et al. 2012; Sun et al. 2018). With more species included, the first divergent lineage also includes *G. dengba*, *G. tibetana*, and *G. dulongensis*. The placement of Middle East and African species is also similar with previous research (Tang, Getahun, and Liu 2009; Yang and Mayden 2010; Zheng, Yang, and Chen 2012). Moreover, species distributed in the same areas do not cluster together but scatteredly distributed on the phylogenetic tree without a clear phylogeography pattern, suggesting a complex evolutionary history.

Myanmar has long been recognized as a region with a high level of freshwater fish diversity. Despite this status, the taxonomy of freshwater fish in Myanmar has been understudied. Zakaria‐Ismail (1994) highlighted that taxonomic review of fishes in Myanmar was insufficient, particularly at the genus level. Despite being one of the biodiversity hot spots, there were only 14 *Garra* species recorded in Myanmar, and many of them were considered widely distributed that need a taxonomic review. The slow pace of species description in Myanmar *Garra* can be attributed to the limited field surveys conducted in this region. Only a handful of new records and descriptions from this region were published for the past two decades. Kullander and Fang (2004) described seven new *Garra* species from Southwest Myanmar. Qin et al. (2017) reported five new record fish species containing two *Garra* species originally described in Yunnan, China. Our species delimitation results suggested that 22 potential species based on molecular and morphological evidence indicating that the diversity of *Garra* in Myanmar has been significantly underestimated. With the current rapid climate change and high extinction rate (Thomas et al. 2004; Urban 2015), more efforts are needed to accelerate the process of new species description, especially in such areas with high level of biodiversity, to better respond to the biodiversity crisis (Sodhi et al. 2004).

### 4.2 Ancestral Distribution of *Garra*

*Garra* was considered to originate from Southeast Asia (Menon 1964). Our ancestral distribution analysis is consistent with this opinion. Previous phylogeny of Labeoninae indicated that genus *Garra* with other genera distributed in Southeast Asia (*Paracrossocheilus*, *Ceratogarra*, *Gonorhynchus*, and *Akrokolioplax*), forming the subtribe Garraina (sensu Yang et al. 2012), further supporting the Southeast Asia origin for *Garra*. With drainage assigned to species, cross‐drainage dispersal events seemed happened multiple times. With drainage assigned to species, cross‐drainage dispersal events seemed happened multiple times. Together with BAMM analysis, the high diversification rate period corresponded to the frequent cross‐drainage dispersal events, suggesting the geological history could contribute to the diversification of *Garra*.

Although the modern Southeast Asian River drainages are largely different with the past, our ancestral distribution reconstruction just support the viewpoint from geology that frequent river rearrangement in Southeast Asia since Miocene (Breitfeld et al. 2022; Jonell et al. 2022). Song et al. (2024) suggested that the elevation of ancient Qinghai‐Tibet Plateau had been reached to about 5000 m before 37 MYA and large rivers originating from Himalayan region gradually formed because of frequent orogeneses since the 23 MYA. High fish diversity in Southeast Asia was attributed to the recent geological movements, especially Himalayan orogeny (He, Cao, and Chen 2001; Peng et al. 2006; Vishwanath 2021). With the continued collision between the Indian plate and Eurasian plate, many mountain streams appeared in Southeast Asia promoting diversification of fish taxa. River system recombination is the main diver of fish vicariance and cross‐drainage distribution (Burridge, Craw, and Waters 2006, 2007; Waters, Burridge, and Craw 2020). Other research in Sisoridae and Schizothoracinae have evidenced that their diversification is related to the river system recombination (Guo, He, and Zhang 2005; He and Chen 2006; Peng et al. 2006). The river systems in Southeast Asia were considered to have experienced multiple recombination and river diversions and become stable until the late Miocene–Pliocene (Jonell et al. 2022; Song et al. 2024). The diversification of *Garra* could be closely linked to the uplift of the Qinghai‐Tibet Plateau and frequent recombination of river systems, providing an explanation for why *Garra* species distributed within the same drainage do not form a monophyletic group.

Due to the limited species coverage in our study, the precise timing and route of *Garra* species dispersal to the Middle East and Africa region remain uncertain. Within the current phylogenetic framework, there are two independent lineages dispersing to the Middle East, one lineage is related to species from South Asia, while the other is related to species from the southern Himalayan region. African species are the result of dispersal of the Middle East species which originate from the South Asia. The result of ancestral distribution reconstruction suggests a complex history of biogeography of *Garra*. Further research with more comprehensive species sampling may provide additional insights into the precise timing and routes of dispersal events of *Garra* species from the Middle East and Africa regions.

## Author Contributions

**Zhi‐Bang Wang:** conceptualization (equal), formal analysis (equal), investigation (equal), methodology (equal), visualization (equal), writing – original draft (equal), writing – review and editing (equal). **Thaung Naing Oo:** conceptualization (equal), data curation (equal), resources (equal). **Lan‐Ping Zheng:** conceptualization (equal), investigation (equal), supervision (equal), writing – review and editing (equal). **Xiao‐Yong Chen:** conceptualization (equal), data curation (equal), funding acquisition (equal), investigation (equal), supervision (equal), validation (equal), writing – review and editing (equal).

## Conflicts of Interest

The authors declare no conflicts of interest.

## Supporting information


Data S1.

## Data Availability

The sequences newly generated in this study have been uploaded to figshare (http://doi.org/10.6084/m9.figshare.25376101).
