# Supplementary material for: Phylogeny, Diversification, and Biogeography of Garra (Cypriniformes: Cyprinidae) Reveals Multiple Cross‐Drainage Dispersals in Southeast Asia
Source: Ecol Evol. 2024 Nov 17;14(11):e70448. doi: 10.1002/ece3.70448 (PMC11569833; doi:10.1002/ece3.70448)
Supplement: Supplementary file 1 — Data S1. [file ECE3-14-e70448-s001.docx]

Table S1 Downloaded species list and access number in Genbank

|  | *COI* | *Cyt b* | *16S* | *RAG1* | *RH* | *EGR2B* | *IRBP* |
| --- | --- | --- | --- | --- | --- | --- | --- |
| *Catostomus catostomus* | NC_037013 | NC_037013 | NC_037013 |  |  |  |  |
| *Myxocyprinus asiaticus* | AB223007 | AB223007 | AB223007 |  |  |  |  |
| *Procypris rabaudi* | NC_011192 | NC_011192 | NC_011192 |  |  |  |  |
| *Cyprinus carpio* | AP009047 | AP009047 | AP009047 |  |  |  |  |
| *Cyprinus megalophthalmus* | NC_028417 | NC_028417 | NC_028417 |  |  |  |  |
| *Cyprinus multitaeniata* | NC_028419 | NC_028419 | NC_028419 |  |  |  |  |
| *Nipponocypris sieboldii* | AB218898 | AB218898 | AB218898 | EU292713 | FJ197069 | FJ531312 | FJ197120 |
| *Labeo stolizkae* | GU086574 | GU086536 | GU168735 | GU086498 | GU086460 | JN160221 | JN160257 |
| *Labeo batesii* | AB238967 | AB238967 | AB238967 | EU711150 | FJ197052 | GQ913576 | FJ197103 |
| *Labeo senegalensis* | AB238968 | AB238968 | AB238968 | EU711151 | FJ197053 | GQ913583 | FJ197104 |
| *Labeo yunnanensis* | JX074205 | JX074282 | JX074123 | JX074486 | JX074619 | JX074534 | JX074573 |
| *Labeo pierrei* | AP011200 | AP011200 | AP011200 | GQ913475 | GQ913526 | GQ913581 | GQ913635 |
| *Labeo forskalii* | JX074210 | JX074287 | JX074128 | JX074491 | JX074624 | JX074539 | JX074577 |
| *Labeo parvus* | JX074208 | JX074285 | JX074126 | JX074489 | JX074622 | JX074537 | JX074575 |
| *G. lissorhynchus* | JX074160 | JX074242 | JX074085 | JX074460 | JX074592 | JX074505 |  |
| *G. spilota* | AP011327 | AP011327 | AP011327 | EU409621 | EU409649 | EU409745 | EU409681 |
| *Garra* sp. *Nepal2* | JX074179 | JX074258 | JX074103 | GQ913466 | GQ913519 | GQ913571 | GQ913626 |
| *G. kempi* | JX074161 | JX074243 | JX074086 | JX074461 | JX074593 | JX074506 |  |
| *G. bicornuta* | JX074156 | JX074238 | JX074081 | JX074456 | JX074588 | JX074501 | JX074549 |
| *G. cf. annandalei* | JX074173 | JX074253 | JX074097 | GQ913460 | GQ913512 | GQ913564 | GQ913619 |
| *G. variabilis* |  | AF180825 |  |  |  |  |  |
| *G. ceylonensis* | JX074172 | JX074252 | JX074096 | GQ913459 | GQ913511 | GQ913563 | GQ913618 |
| *G. mullya* | JX074155 | JX074237 | JX074080 | JX074455 | JX074587 | JX074500 | JX074548 |
| *G. gotyla* | JX074177 | JX074256 | JX074101 | GQ913463 | GQ913515 | GQ913567 | GQ913622 |
| *G. fuliginosa* | JX074176 | JX074255 | JX074100 | GQ913462 | GQ913514 | GQ913566 | GQ913621 |
| *G. orientalis* | HM536884 | HM536788 | DQ845884 | GQ913464 | GQ913516 | GQ913568 | GQ913623 |
| *G. mirofrontis* | GU086603 | GU086565 | GU168764 | GU086527 | GU086489 | JN160215 | JN160251 |
| *G. cryano* | JX074214 | JX074291 | JX074132 |  |  |  |  |
| *G. nasuta* | JX074219 | JX074295 | JX074137 | JX074494 | JX074627 | JX074543 | JX074581 |
| *G. lamta* | JX074158 | JX074240 | JX074083 | JX074458 | JX074590 | JX074503 | JX074551 |
| *G. makiensis* |  | JX097082 |  | GQ911677 | GQ911685 | GQ911675 | GQ911663 |
| *G. rufa* | AP011197 | AP011197 | AP011197 | JX074472 | JX074605 | JX074520 | JX074563 |
| *G. barreimiae* |  | EU000504 |  |  |  |  |  |
| *G. waterloti* | JX074212 | JX074289 | JX074130 | JX074493 | JX074626 | JX074541 | JX074579 |
| *G. congoensis* | JX074174 | JX074254 | JX074098 | GQ911679 | GQ911687 | GQ911669 | GQ911666 |
| *G. ornata* | JX074202 | JX074279 | JX074120 | JX074483 | JX074616 | JX074531 |  |
| *G. tana* |  | FJ196827 |  |  |  |  |  |
| *G. qiaojiensis* | JQ864604 | JQ864583 |  | JQ864623 |  |  | JQ864641 |
| *G. bispinosa* | JQ864615 | JQ864594 |  |  |  |  |  |
| *G. rotundinasus* | JQ864616 | JQ864595 |  | JQ864634 |  |  | JQ864651 |
| *G. salweenica* | JQ864614 | JQ864593 |  | JQ864633 |  |  | JQ864650 |
| *G. surgifrons* | KX983934 | KX983937 |  | KX983944 |  |  | KX983940 |
| *G. longchuanensis* |  | KT959359 |  |  |  |  |  |
| *G. dengba* |  | MH243435 |  |  |  |  |  |
| *G. motuoensis* |  | MG490162 |  |  |  |  |  |
| *G. tibetana* | MG999836 | MG999836 | MG999836 |  |  |  |  |
| *G. yajiangensis* |  | MG490159 |  |  |  |  |  |
| *G. dulongensis* | JQ864611 | JQ864590 |  | JQ864630 |  |  | JQ864647 |

Table S2 distribution area of each species and code.

| Sample name | Distribution area | code | Sample name | Distribution area | code |
| --- | --- | --- | --- | --- | --- |
| *G. tengchongensis* | Irrawaddy River basin | A | *G. ngatangka* CXY20190563 | Irrawaddy River basin | A |
| *G. dengba* | Yarlung Zangbo River basin | B | *G. poecilura* CXY20170137 | Irrawaddy River basin | A |
| *G. tibetana* | Yarlung Zangbo River basin | B | *G. poecilura* CXY20190561 | Irrawaddy River basin | A |
| *G. dulongensis* | Irrawaddy River basin | A | *G. poecilura* CXY20190430 | Irrawaddy River basin | A |
| *G. dulongensis* CXY20150171 | Irrawaddy River basin | A | *G. bicornuta* | India Peninsula | B |
| *G. nigricollis* CXY20190401 | Kaladan River basin | A | *G. cf. annandalei* | India Peninsula | B |
| *Garra* sp3 CXY20190325 | Kaladan River basin | A | *G. variabilis* | the Middle east | C |
| *G. cf. ukhrulensis* CXY20190324 | Kaladan River basin | A | *G. mullya* | India Peninsula | B |
| *G. chivaensis* CXY20190410 | Irrawaddy River basin | A | *G. ceylonensis* | India Peninsula | B |
| *Garra* sp4 QT20180043 | Irrawaddy River basin | A | *G. cf. gravelyi* CXY20190483 | Irrawaddy River basin | A |
| *Garra* sp. Nepal2 | Yarlung Zangbo River basin | B | *G. nasuta* | Yarlung Zangbo River basin | B |
| *G. kempi* | Yarlung Zangbo River basin | B | *G. lamta* | Yarlung Zangbo River basin | B |
| *G. spilota* CXY20190539 | Irrawaddy River basin | A | *G. barreimiae* | the Middle east | C |
| *G. spilota* | Irrawaddy River basin | A | *G. rufa* | the Middle east | C |
| *Garra* sp5 QT20180075 | Irrawaddy River basin | A | 20190237 *G. cf. salweenica* | Salweenica River basin | A |
| *G. manipurensis* CXY20190403 | Kaladan River basin | A | *Garra* sp2 CXY20190559 | Irrawaddy River basin | A |
| *G.manipurensis* CXY20190342 | Kaladan River basin | A | *G. gravelyi* QT20180127 | Salweenica River basin | A |
| *G. flavatra* | Kaladan River basin | A | *G. cf. qiaojiensis* CXY20160301 | Irrawaddy River basin | A |
| *G. rakhinica* CXY20190376 | Kaladan River basin | A | *G. bispinosa* | Irrawaddy River basin | A |
| *G. matensis* CXY20190314 | Kaladan River basin | A | *G. qiaojiensis* S20180480 | Irrawaddy River basin | A |
| *G. lissorhynchus* | Yarlung Zangbo River basin | B | *G. qiaojiensis* | Irrawaddy River basin | A |
| *G. ceylonensis* | India Peninsula | B | *G. longchuanensis* | Irrawaddy River basin | A |
| *G. cf. gravelyi* CXY20190483 | Irrawaddy River basin | A | *G. tana* | Africa | D |
| *G. nasuta* | Yarlung Zangbo River basin | B | *G. waterloti* | Africa | D |
| *G. lamta* | Yarlung Zangbo River basin | B | *G. ornata* | Africa | D |
| *G. barreimiae* | the Middle east | C | *G. congoensis* | Africa | D |
| *G. rufa* | the Middle east | C | *G. surgifrons* | Irrawaddy River basin | A |
| *G. makiensis* | Africa | D | *G. fuliginosa* | Chao Phraya &Mekong River basin | A |
| *G. tana* | Africa | D | *G. salweenica* | Salweenica River basin | A |
| *G. waterloti* | Africa | D | *G. orientalis* | South China | E |
| *G. ornata* | Africa | D | *G. mirofrontis* | Mekong River basin | A |
| *G. congoensis* | Africa | D | *G. cyrano* | Mekong River basin | A |
| *G. surgifrons* | Irrawaddy River basin | A | *G. yajiangensis* | Yarlung Zangbo River basin | B |
| *G. nasuta* | Yarlung Zangbo River basin | B | *G. rotundinasus* | Irrawaddy River basin | A |
| *G. lamta* | Yarlung Zangbo River basin | B |  |  |  |
| *G. barreimiae* | the Middle east | C |  |  |  |
| *G. rufa* | the Middle east | C |  |  |  |
| *G. makiensis* | Africa | D |  |  |  |
| *G. motuoensis* | Yarlung Zangbo River basin | B |  |  |  |
| *G. gotyla* | Yarlung Zangbo River basin | B |  |  |  |
| *G. qiaojiensis* QT20170223 | Irrawaddy River basin | A |  |  |  |

Table S3 Result of biogeographic model test with detailed categories. The best model was highlighted in bold.

| Model | LnL | Numbers of parameters | parameters | | AICc | | AICc_wt | |
| --- | --- | --- | --- | --- | --- | --- | --- | --- |
|  |  |  | d | e |  |  |  |  |
| DEC | -141.5 | 2 | 0.0034 | 0.010 | | 287.3 | | 0.0033 |
| **DIVALIKE** | **-135.8** | **2** | **0.0036** | **0.0022** | | **275.8** | | **1.00** |
| BAYAREALIKE | -155.4 | 2 | 2 | 0.0048 | | 315 | | 3.1e-09 |


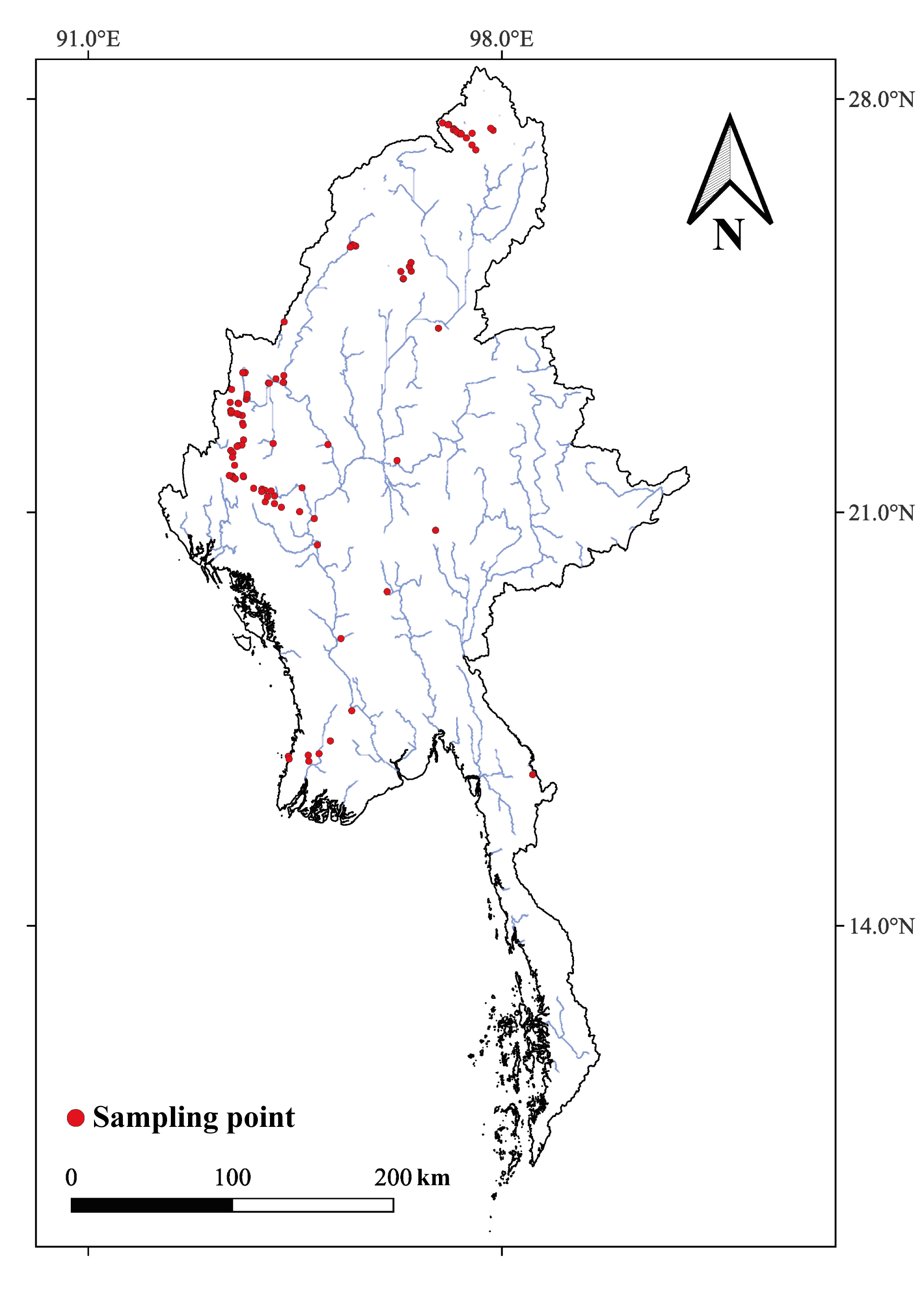


Figure S1 Myanmar map showing the location of *Garra* specimens sampled in this study.


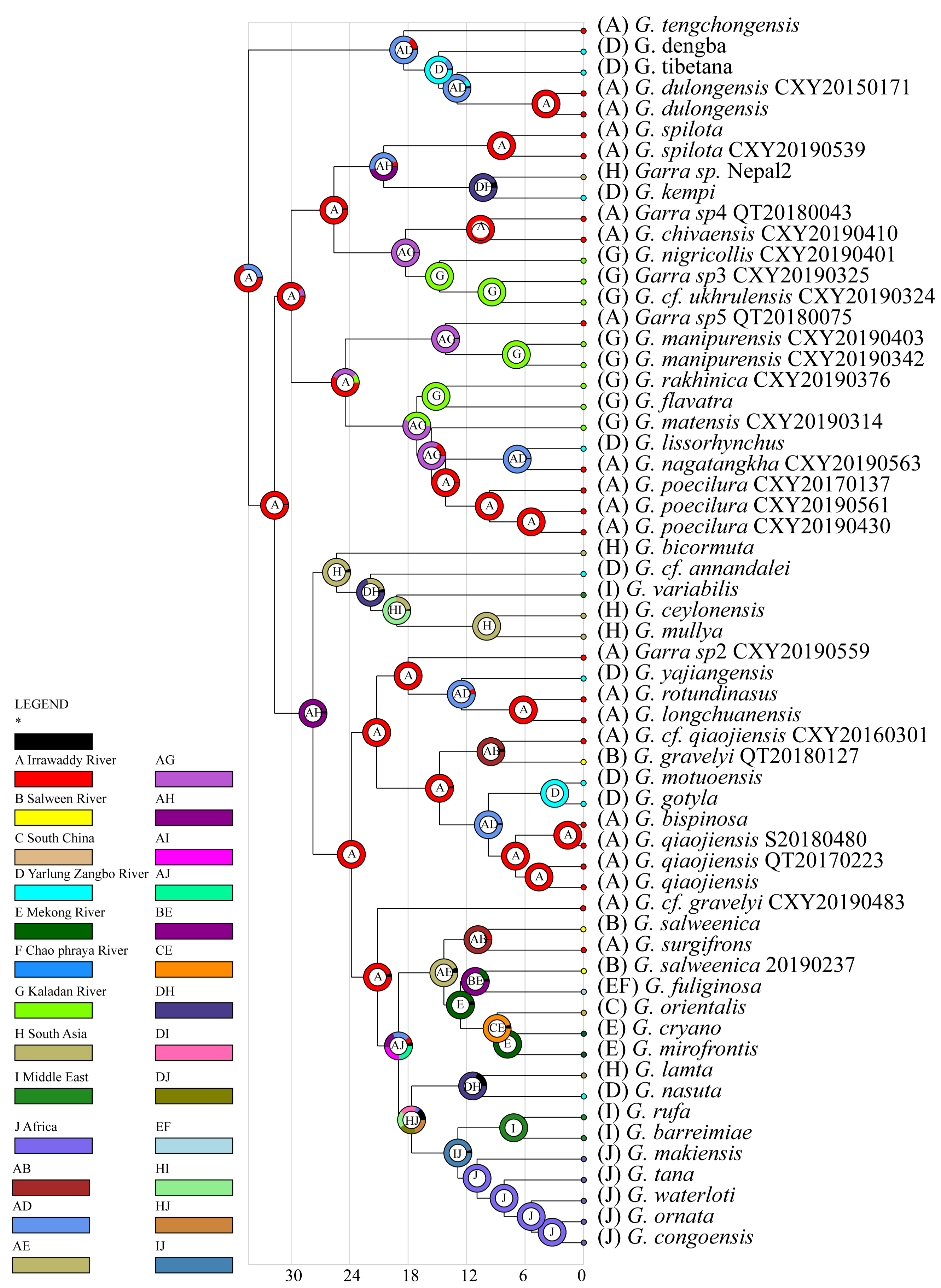


Figure S2 Reconstruction of ancestral distribution with detailed categories. A: Irrawaddy River; B: Salween River; C: South China; D: Yarlung Zngbo River; E: Mekong River; F: Chao Phraya River; G: Kaladan River, H: South Asia, I: Middle East, J, Africa.
